# Improving access to new diagnostics through harmonised regulation: priorities for action

**DOI:** 10.4102/ajlm.v3i1.123

**Published:** 2014-04-04

**Authors:** Ruth McNerney, Kimberly Sollis, Rosanna W. Peeling

**Affiliations:** 1 London School of Hygiene & Tropical Medicine, United Kingdom

## Abstract

A new generation of diagnostic tests is being developed for use at the point of care that could save lives and reduce the spread of infectious diseases through early detection and treatment. It is important that patients in developing countries have access to these products at affordable prices and without delay. Regulation of medical products is intended to ensure safety and quality whilst balancing the need for timely access to beneficial new products. Current regulatory oversight of diagnostic tests in developing countries is highly variable and weak regulation allows poor-quality tests to enter the market. However, inefficient or overzealous regulation results in unnecessary delays, increases costs and acts as a barrier to innovation and market entry. Setting international standards and streamlining the regulatory process could reduce these barriers. Four priority activities have been identified where convergence of standards and protocols or joint review of data would be advantageous: (1) adoption of a common registration file for pre-market approval; (2) convergence of quality standards for manufacturing site inspections; (3) use of common evaluation protocols, as well as joint review of data, to reduce unnecessary duplication of lengthy and costly clinical performance studies; and (4) use of networks of laboratories for post-market surveillance in order to monitor ongoing quality of diagnostic devices. The adoption and implementation of such measures in developing countries could accelerate access to new diagnostic tests that are safe and affordable.

## Introduction

Diagnostic tests make a major contribution to global health. They are needed in order to guide treatment decisions and ensure the appropriate use of medicines. They are also vital for screening for infections, such as HIV infection or syphilis, in asymptomatic individuals.^1^ Such tests are often lifesaving, where delay or lack of access can result in deterioration of the patient’s health and lead to further complications. Diagnostics are particularly important for the control of infectious diseases, where early detection allows intervention in order to prevent onward transmission.^2^ They may also be used to monitor the outcome of treatment. Most diagnostic interventions utilise *in vitro* diagnostic medical devices (IVDs) which test specimens obtained from a patient, such as blood or urine. IVDs include a wide range of technologies, from rapid dip-stick strips for use at the point of care (POC) to sophisticated instrumentation for use in referral laboratories. Access to diagnostic tests in developing countries is often limited by their availability, as patients frequently do not live within easy travelling distance of a well-equipped and functional laboratory.^2,3^ A new generation of diagnostic tests is being developed for use at the point of care that will not need a laboratory.^4,5^ Prompt access to the new tests should be encouraged; if they are made available in developing countries at affordable prices, such tests could increase access to appropriate healthcare, thereby saving lives and reducing the spread of infectious diseases.^4,5,6^

### Regulating diagnostic devices

Regulation of medical products is intended to ensure safety and quality whilst ensuring that the public has timely access to beneficial new products. National Regulatory Authorities (NRAs) are usually mandated by law and, for a regulatory system to be effective, it should be enforceable in both the public and private healthcare sector. Following impartial review, new tests that are considered substandard or unsafe are refused entry to the market, whereas satisfactory products are approved and registered. For regulatory purposes, IVDs are classified as medical devices. In this context, ‘medical device’ means any instrument, apparatus, implement, machine, appliance, implant, reagent for *in vitro* use, software, material, or any other similar or related article intended by the manufacturer to be used alone or in combination, for human beings for one or more of the specific medical purpose(s): diagnosis, prevention, monitoring, treatment or alleviation of disease; diagnosis, monitoring, treatment, alleviation of or compensation for an injury; investigation, replacement, modification or support of the anatomy or of a physiological process; support or sustenance of life; control of conception; disinfection of medical devices; provision of information by means of *in vitro* examination of specimens derived from the human body.^7^ An IVD is defined as:

medical device, whether used alone or in combination, intended by the manufacturer for the *in vitro* examination of specimens derived from the human body solely or principally to provide information for diagnostic, monitoring or compatibility purposes.^7^

Most countries have a legal framework and a nominated body to regulate medicines, but regulation of medical devices in developing countries is less common.^8^ The demands on regulatory authorities for IVDs differ from that of other medical products in that there are a very large number of IVDs and, compared with drugs or vaccines, their market life is often short given the rapid pace of technology development.^9^ Current regulatory oversight of IVDs is variable^8^ and, in countries where there is no regulation, substandard and counterfeit tests may be sold openly.^10^ In countries that *do* regulate, approval for IVDs is often costly, lengthy and, on occasion, lacking in transparency, thus regulation of diagnostics is currently seen as a barrier to innovation and access.^9,11,12^ In 2001, the Pan American Health Organisation (PAHO), the Regional Office for the Americas of the World Health Organization (WHO) and the United States Food and Drug Administration (USFDA) published a model regulatory programme for medical devices, with a set of guiding principles.^13^ These principles include the statement that:

regulatory system should ensure that valuable new technologies are made available to the clinical community and to patients and consumers expeditiously while preventing unsafe or ineffective devices from reaching the market.^13^

They also state that regulatory decisions must be based on strong and clear science, free of external influences and, in addition, that countries instituting regulatory programmes should be cognisant of ongoing international efforts to harmonise activities.

Regulatory control of IVD has three components:

- *Pre-market evaluation*: to assess safety, performance, benefits and risks prior to approval to market the device.
- *Marketing controls*: to stipulate conditions under which devices can be offered for sale; identify who may use the device and under what conditions; and to avoid inappropriate marketing or misleading claims regarding test effectiveness.
- *Post-marketing controls*: to maintain vigilance and continued safety and quality of approved products that entails a method for information-sharing amongst users within the country and across national health authorities. There should be a means by which corrections can be made as well as a mechanism for removing substandard products that pose a risk to public health.

### Risk classification of *in vitro* diagnostic medical devices

The degree of regulatory control for a medical device should be proportionate to the risk posed by the product. Unlike medicines or vaccines, IVDs are not ingested by the patient, greatly reducing the potential for harm. Thus the stringency of regulatory oversight required is related to the harm that a false positive or false negative test result may cause to either individual or public health. Reagents used in diagnostics tests, such as microbiological stains or culture media, by themselves pose little risk to human or public health and are classed as low risk. High risk tests include those used to screen for infections such as HIV, where a false negative test result could lead to the individual not being given life-saving drugs and continuing to transmit the infection within the community. Should the test be used to screen blood products, then recipients of that donation would be at risk of acquiring a life-threatening disease. Such tests require more stringent control, including evidence of their performance as obtained through clinical studies. To guide the regulatory process, products are grouped or classified according to their risk of causing harm. In 2006, the Global Harmonization Task Force (GHTF), a voluntary partnership of stringent regulatory authorities and diagnostic companies, published recommendations regarding classification of medical devices. They suggested that each medical device be assigned to one of four classes based upon its intended use (Table 1).^14^ Classification provides a mechanism by which the cost and delay of registration are moderated according to the potential of a product to cause harm. Manufacturers are required to provide less-substantial submission dossiers for products in risk group Class A; whereas Class D products require stringent conformity assessment, including evidence of performance in a clinical setting that is representative of the intended use. For devices to be used at the POC, studies should be conducted in the settings of intended use (clinic or outreach settings) with testing performed by local health providers. Such studies are costly and, for some diseases, may require years to plan and execute. The classification system requires a shorter and less-costly route to pre-market approval for low-risk products.

**TABLE 1:** Classification of medical devices with examples of diagnostic products.

Class	Risk Level	Examples
A	Low individual risk and low public health risk	Stains, culture reagents
B	Moderate individual risk and/or low public health risk	Home-use pregnancy tests, urine test strips
C	High individual risk and/or moderate public health risk	Rapid tests for rubella, malaria
D	High individual risk and high public health risk	Blood screening tests for HIV, Hepatitis B and C, or Human T-cell lymphotropic virus

### Harmonising regulation of diagnostic devices

Whilst regulation of medical products is required in order to ensure their safety, it is essential that regulatory review processes do not obstruct or unnecessarily delay access to beneficial new products. It is recognised that the current lack of standardisation across national regulatory authorities and the lack of clarity surrounding the regulatory pathways presents an unnecessary burden on manufacturers and acts as a deterrent to marketing in countries where the financial returns may be modest.^9^ With the exception of the countries of the European Union, where a harmonised system has been adopted, countries each have their own set of requirements. In some countries, transparency is lacking, with little information available about the regulatory process or the fees charged. Several transnational initiatives are striving toward improved harmonisation of regulation of medical devices, including IVDs (Table 2). Harmonisation requires the use of standardised terminology and definitions which are employed to classify the products under regulation. Guidance on this topic was issued by the GHTF, which transitioned to the International Medical Device Regulators Forum (IMDRF) in 2012.

**TABLE 2:** Organisations promoting regulatory harmonisation of medical devices or *in vitro* diagnostics.

Name	Regional focus	Membership
Global Harmonisation Task Force (GHTF)*	Global	NRAs and manufacturers. Founding Members: Australia, Canada, the European Union, Japan and the United States
International Medical Device Regulators Forum (IMDRF)	Global	NRAs: Australia, Canada, the European Union, Japan and the United States. China and the Russian Federation to be confirmed. WHO has observer status
Asian Harmonisation Working Party (AHWP) www.ahwp.info	Asia	23 member economies: regulators and companies
Latin America IVD Association (ALADDIV)	Latin America	Forum for regulators, researchers, laboratory experts and representatives from ministries of health
Pan-African Harmonisation Working Party (PAHWP) www.pahwp.org	Africa	African regulators, laboratory experts, manufacturers and international organisations

* Disbanded 2012 and reformulated as IMDRF; NRA, national regulating authority; WHO, World Health Organization.

For IVDs used in developing countries there are opportunities to streamline and harmonise activities where convergence of protocols and mutual recognition of other regulatory bodies could improve their safety and quality, accelerating access to new tests while simultaneously minimising the costs incurred. Setting international standards and streamlining the regulatory process could reduce the regulatory burden and lower costs of new products. Four priority areas for harmonisation have been recognised: (1) adoption of common registration requirements and submission dossiers for pre-market approval; (2) convergence of quality standards and mutual, or third party, recognition of audit activities; (3) rationalisation of clinical performance studies in order to avoid unnecessary duplication; and (4) building of transnational networks for post-market surveillance. Each of the aforementioned areas of harmonisation is discussed in the following sections. The status quo and need for change are described, along with recommendations made for the way forward. The impact of the proposed harmonisation activities are summarised in Table 3.

**TABLE 3:** Expected impact of harmonisation activities.

Item	Status quo	Proposed harmonisation model	Impact
Dossier for registration	Forms unique to each country	Common template submission	Standard dossier conforming to international standards whilst saving companies time, effort and expense
Quality systems audit	ISO 13485 Unique visits by NRAs; delay in approval due to long queues and high costs to companies	Adoption of common standard Mutual recognition of audits Recognition of third party audits by an ‘accredited’ body	Shortened times to approval and reduced costs Faster access to tests for patients
Clinical studies	Large number of studies conducted for each product	Common protocols Network of competent sites Joint review of data but final approval country specific	Approval in more countries with fewer trials Clear path for approval; reduced costs and time to approval yielding improved access by patients
Post-marketing surveillance	Limited capacity for identifying low quality products and product failures	Network of evaluation sites for post-marketing surveillance	Ensured quality of tests post-approval

## A Common Submission Dossier

Companies seeking approval to market an IVD are required to supply a dossier to the appropriate NRA describing the device and documenting evidence relating to the quality of manufacture, as well as the safety and stability of the components. In addition, those devices considered to be at risk of causing harm to either individual or public health also require evidence regarding the clinical performance of the device. The adoption of a standardised submission dossier template would promote efficiency within the regulatory review process and facilitate standardised training on good review practice using a common set of teaching materials. NRAs would retain independent review of the data, along with any decisions for approval based on national requirements as they pertain to their local population needs. The need for a common submission dossier was one of the top priorities recognised by the GHTF and was an activity already adopted by the Asian Harmonisation Working Party (AHWP).^15^

### Status quo

With the exception of the European Union, submission dossiers are unique to the country, with each NRA utilising its own indicators, nomenclature and format in its own language.^9,16^ Preparation of a submission dossier for regulatory approval is a substantial undertaking and the necessity of preparing individual dossiers and reformulating data for each NRA is an unnecessary burden on diagnostic companies, causing overall increase to the cost of marketing a new product. In countries with weak economies, small populations or low prevalence of the condition or disease to be tested, the anticipated market may be insufficient to warrant the cost of registering the product. The problem is particularly acute for small companies with limited regulatory expertise or capacity. Large companies are also not exempt from the burden that a submission dossier carries, as it represents a significant commitment of resources and time. A standardised template for submission dossiers would decrease the time and effort required of companies, improve the efficiency of regulatory reviews and reduce the cost of goods for diagnostic companies, resulting in a more affordable product and overall reduced time to market.

### Action going forward

Following principles established by the GHTF, the AHWP has developed a Common Submission Dossier Template (CSDT) for premarket submission of IVDs. The dossier incorporates: (1) marketing history and, where appropriate, a risk/benefit assessment, any prior approvals and current regulatory status, as well as documentation to demonstrate conformity to the essential principles of the GHTF; (2) a product description which includes intended use and any warnings or precautions; (3) a summary of design verification and validation documents – these may include sensitivity, specificity, precision, stability, storage and controls – and, depending on the product classification, it may also include evidence from clinical performance studies; (4) device labelling, with instructions for use (including operating manual and user manual), patient information leaflet and promotional materials; (5) a summary of the risks identified and a description of how these risks have been controlled to an acceptable level; and (6) manufacturer information in order to identify manufacturing sites and to provide Quality Management System certification such as (ISO) 13485:2003 and a description of the manufacturing process.^15^ A common dossier is to be piloted in Africa and other regions using a POC test as an example. Transparency of regulatory requirements and processes shall be promoted, preferably with documents available on line. Good Review Practice will include the monitoring and publication of the time required for review. Fees charged to companies should reflect the costs of the approval process and should not be seen as an opportunity for profit. Harmonisation of submission dossier requirements and adoption of a common template will: (1) reduce the cost to manufacturers of registering a product, a cost that would ultimately be passed to the consumers of the test; (2) reduce delays in test registration; (3) reduce barriers to marketing in small economies; and (4) facilitate harmonised approval of diagnostic devices, ultimately reducing the burden on NRAs and the cost of national regulatory approval. Harmonisation will require consensus on the fundamental principles of regulating diagnostic devices for health, but independent decision making shall be retained by the NRA.

## Convergence in the auditing of quality systems

Regulatory oversight requires assurance that the manufacturer of the device conforms to a satisfactory quality management system. These quality management standards are universal and should not differ from country to country. The quality management system used by a manufacturer to control the quality of their product should be audited in order to provide assurance that safe and effective devices will be manufactured. The audit should make certain that specified minimum standards are met during manufacturing and provide impartial, reliable and objective evaluation of compliance with regulatory requirements. ISO 13485:2003 is an international standard established for the manufacture of IVDs.^17^ If satisfactory quality management is not practised by the manufacturers, corrective measures may be recommended. For IVDs considered to have a risk of causing harm (to potential patients or users), assessment of adherence to satisfactory quality systems will include visiting the site of manufacture. Audit teams must encompass a range of skills and expertise enabling them to assess the quality management system and to determine the effectiveness of its implementation. The range of skills includes understanding the regulations and standards applicable to the specific IVD submitted for approval, the intended use and associated risks of the device, as well as the knowledge to assess the design, manufacturing processes and technologies involved. Convergence of quality standards and mutual or third-party recognition of inspection results could reduce duplication of efforts where teams representing different NRAs undertake independent audit of the same site, saving expenditure and reducing delays in regulatory approval.

### Status quo

Regulatory audits and site visits are costly for companies, with each audit costing as much as USD 200 000 (personal communication). Although costs are met in the first instance by the companies, they will ultimately be reflected in the price of the product and recouped from the end users. Duplication of audits inflates the cost of bringing a product to market and results in unnecessary delays. NRAs that require manufacturing site inspections for every medical device sold often have long delays in approving products for the market. In their 2012 annual report, the Brazilian National Health Surveillance Agency (ANVISA), reported a backlog of over 1000 products awaiting evaluation.^18^ NRAs in the developing world lack the expertise and capacity required in order to undertake audits, which ultimately results in delayed approval.

### Action going forward

Minimum standards should be identified for quality management in the manufacture of POC diagnostics in addition to ISO 13485:2003.^17^ To reduce duplication, competent authorities or organisations capable of undertaking inspections should be identified and mutual recognition or recognition of competent third-party quality management audits adopted. In addition, mechanisms for sharing information should be established.

Harmonisation of quality systems audits through convergence of standards and/or mutual recognition will make more efficient use of auditing resources and reduce the number of audits by different regulatory bodies for the same product, saving costs and reducing delays in new products reaching the market. Use of standardised audit protocols and expert bodies will streamline auditing, leading to improved quality management and product quality. This will provide greater consistency and increased confidence in audits. Enhanced consistency in audit practices and feedback provided to manufacturers regarding quality management will facilitate improvements in manufacturing practice. The ultimate beneficiaries will be patients and users of diagnostic devices, who will gain an increased assurance that medical devices placed on the market are safe and effective whilst simultaneously ensuring that the costs of implementing an effective system do not unnecessarily inflate the price.

## Reducing duplication in studies of clinical performance

Pre-market approval of those IVDs considered to be high risk to individual or public health requires supporting evidence of the performance and operational characteristics of the device. For laboratory-based diagnostic tests, such evaluations are often conducted using well-characterised archived samples. If the new product is a POC test intended for use in decentralised health centres or dispensaries, evaluations of sensitivity, specificity, precision and ease of use should be conducted in the settings of intended use, with the test performed by the proposed end users.^19^ Such studies need to be based on sufficient sample size with assurance of quality so as to allow informed decisions on the performance and utility of the device as to whether the probable benefits of the device outweigh the risks. Data generated in clinical studies are presented to NRAs as part of the submission dossier for pre-market approval. The high cost of clinical trials and the length of time they require may result in higher cost of goods and significant delay in gaining access to new products that could potentially save lives.^20^

### Status quo

The requirement of NRAs to have national trial data for approval has resulted in unnecessary duplication, where further studies provide little or no added scientific benefit.^21,22^ High costs and delays incurred during clinical studies are a deterrent for companies entering the market, particularly in smaller countries where financial returns may be modest.^9,12^ Costs borne by the manufacturer are ultimately passed on to the consumer, making devices less affordable. Since not every country has the capacity to conduct high quality studies in a timely manner, resulting from a lack of specialist facilities or limited access to appropriate patient groups, multi-country evaluations and NRAs coming together to review trial data and then making their own decisions for approval would be a more efficient approach.

### Action going forward

Multi-country studies using a standardised protocol and joint review of trial data should be encouraged in order to shorten trial duration and reduce duplication. A clinical trials registry should be established to reduce unintentional duplication and promote maximal use of resources. Clinical performance studies should be conducted by accredited laboratories and clinics with external oversight, so as to ensure conformity with international standards, including good laboratory and clinical practice.^23,24,25^ Prior joint approval of clinical and laboratory protocols by institutional review boards will ensure quality and acceptance by representatives of regulatory authorities.

Reducing duplication of clinical trials will: (1) reduce the cost of accessing the market, making tests more affordable; (2) reduce delays in regulatory approval, accelerating access to new products; (3) reduce barriers to marketing in small economies; and (4) allow NRAs to maintain independent decision making for new products without requiring local trials.

## Convergence in post-market surveillance

Post-market surveillance ensures that products continue to meet expected safety and quality standards following approval by NRAs and an important component of regulatory oversight of diagnostic products for health.^26^ Proactive post-market surveillance requires the systematic collection of data from laboratory studies in order to monitor test performance using a panel of appropriate reagents or, alternatively, through field testing using a panel of well-characterised samples. Reactive surveillance requires manufacturers, or testers, to report problems voluntarily through an established reporting system. Such activities are applicable to manufacturers, importers and distributors.

There is a need for cross-border, regional and global sharing of adverse events that threaten safety of individuals or public health so as to accelerate information gathering and enable substandard products to be withdrawn more quickly. The National Competent Authorities Report (NCAR) is a membership-based system open to those countries with stringent regulatory authorities.^27^ The system incorporates post-market surveillance, vigilance and reporting of adverse events and is aimed at improving safety by reducing the likelihood of repeated adverse events. There are two levels of NCAR participation. Full participation involves national competent authorities with established national adverse event reporting programmes. Being a full participant, a national authority receives both public and confidential or highly-sensitive information from other NCAR participants. Associate participants are a second tier of membership that receive public information, such as recall notices, safety and hazard alerts.

### Status quo

Currently, there is limited capacity for post-market surveillance in much of the developing world. Systems for post-market surveillance for diagnostic devices are not implemented globally^26^ and, in most developing countries, reporting and information sharing occurs on an *ad hoc* basis. In many countries, random quality checks, such as lot testing, are not performed and tests can enter the market without checks on their quality.^21,28^ Most developing countries lack a feedback mechanism to provide manufacturers with information regarding the need for corrective action as well as a mechanism for removal of substandard tests that present a risk to public health. Disruption of services for some priority diseases, such as HIV, has occurred where publicity regarding quality problems has led, in some countries, to discontinuation of use without replacement devices being available.^29^

### Action going forward

A mix of proactive and reactive post-market surveillance activities should be encouraged. Reporting should be mandatory in the case of a death, a serious injury that is life threatening or results in permanent damage or impairment of a body function, or a malfunction. To implement post-market surveillance for IVD medical devices in developing countries, regional networks of accredited laboratories should be established in order to undertake batch testing and monitor performance and safety. Standardised protocols should be established and reference quality assurance materials shared. These practices would promote good practice and instil confidence in the system, in addition to facilitating the exchange of data.

Global and regional mechanisms should be established so as to facilitate investigation and procedural corrective actions or recalls for products found to be unsatisfactory. Harmonisation of proactive activities, such as batch monitoring, would enhance safety by reducing reporting delays, allowing prompt action to be taken should unsafe products be reported. Standardisation of protocols, test reagents and sample panels across geographic regions would assist surveillance whilst simultaneously minimising costs. National and transnational communication platforms are needed to simplify current procedures. Networks of accredited laboratories should be established to facilitate mutual recognition of surveillance data.

Convergence of post-market surveillance activities will: (1) ensure that products continue to meet expected safety, performance and efficacy requirements following pre-market approval; (2) prevent substandard tests, or batches of tests, from entering the market; (3) enable corrective actions to be taken by manufacturers; and (4) facilitate removal of substandard tests from the market, ultimately reducing costs and maximising efficient use of resources by NRAs.

## Conclusion

Diagnostic tests can play a vital role in improving access to effective healthcare, but the current regulatory landscape in developing countries creates significant barriers to market entry and has become a deterrent to innovation. Setting international standards and streamlining the regulatory process for *in vitro* diagnostic devices could diminish the regulatory burden. Four areas where action could lower costs, reduce delays, and accelerate access to quality diagnostic products for health are: (1) adoption of common registration requirements and submission dossier for pre-market approval; (2) convergence of quality standards and mutual, or third-party, recognition of audit activities; (3) rationalisation of clinical performance studies in order to avoid unnecessary duplication; and (4) building transnational networks for post-market surveillance.
